# O.4.2-1 The within-person bidirectional association between physical activity and loneliness in the daily lives of adolescents and young adults

**DOI:** 10.1093/eurpub/ckad133.176

**Published:** 2023-09-11

**Authors:** Lise Jennen, Victor Mazereel, Kristof Vansteelandt, Claudia Menne-Lothmann, Jeroen Decoster, Catherine Derom, Evert Thiery, Bart P F Rutten, Nele Jacobs, Jim van Os, Marieke Wichers, Marc De Hert, Davy Vancampfort, Ruud van Winkel

**Affiliations:** Department of Neurosciences, Center for Clinical Psychiatry, KU Leuven, Leuven, Belgium; Department of Neurosciences, Center for Clinical Psychiatry, KU Leuven, Leuven, Belgium; University Psychiatric Center KU Leuven, Leuven-Kortenberg, Belgium; University Psychiatric Center KU Leuven, Leuven-Kortenberg, Belgium; Department of Psychiatry and Neuropsychology, School of Mental Health and Neuroscience, Maastricht University, Maastricht, The Netherlands; University Psychiatric Centre Sint-Kamillus, Bierbeek, Belgium; Department of Obstetrics and Gynecology, Ghent University Hospital, Ghent University, Ghent, Belgium; Department of Neurology, Ghent University Hospital, Ghent University, Ghent, Belgium; Department of Psychiatry and Neuropsychology, School of Mental Health and Neuroscience, Maastricht University, Maastricht, The Netherlands; Department of Psychiatry and Neuropsychology, School of Mental Health and Neuroscience, Maastricht University, Maastricht, The Netherlands; Faculty of Psychology, Open University of the Netherlands, Heerlen, The Netherlands; Department of Psychiatry and Neuropsychology, School of Mental Health and Neuroscience, Maastricht University, Maastricht, The Netherlands; Department of Psychosis Studies, Institute of Psychiatry, King’s Health Partners, King’s College London, London, UK; Department Psychiatry, Brain Center Rudolf Magnus, University Medical Centre Utrecht, Utrecht, The Netherlands; University of Groningen, University Medical Center Groningen, Interdisciplinary Center Psychopathology and Emotion Regulation, Groningen, The Netherlands; Department of Neurosciences, Center for Clinical Psychiatry, KU Leuven, Leuven, Belgium; University Psychiatric Center KU Leuven, Leuven-Kortenberg, Belgium; Department of Neurosciences, Center for Public Health Psychiatry, KU Leuven, Leuven, Belgium; Antwerp Health Law and Ethics Chair – AHLEC University Antwerp, Antwerp, Belgium; University Psychiatric Center KU Leuven, Leuven-Kortenberg, Belgium; Department of Rehabilitation Sciences, KU Leuven, Leuven, Belgium; lise.jennen@kuleuven.be; Department of Neurosciences, Center for Clinical Psychiatry, KU Leuven, Leuven, Belgium; University Psychiatric Center KU Leuven, Leuven-Kortenberg, Belgium; Department of Neurosciences, Center for Public Health Psychiatry, KU Leuven, Leuven, Belgium

## Abstract

**Purpose:**

Adolescents and young adults are at high risk for experiencing loneliness, a well-established risk factor for mental health symptoms. Previous cross-sectional studies show an inverse association between loneliness and physical activity. The causality and directionality of this association remains unclear as longitudinal and interventional studies are currently lacking in this population. With the Experience Sampling Method, we are able to investigate fluctuations in daily life and within-person dynamics. We hypothesized a bidirectional relationship where physical activity can reduce loneliness and loneliness can reduce physical activity the subsequent moment.

**Methods:**

Participants were sampled from the population-based twin registry TwinssCan. The sample consisted of 784 participants, aged 15 to 25 years. They responded to a questionnaire ten times a day for six days. Analysis was done with multilevel models. Interaction effects with affective valence (enjoyment, competence and effort), and depressive and anxiety symptoms were explored.

**Results:**

We did not observe a significant association between physical activity and loneliness on the within-person level, but there was a significant interaction effect where physical activity predicts decreased subsequent loneliness, but only at moments with high affective valence where young people enjoy the activity, feel competent and requires limited effort. There was no significant interaction effect with depressive and anxiety symptoms.

**Conclusions:**

The findings of this study give us a better understanding of the association between physical activity and loneliness in young people. During the presentation, we will discuss the importance of considering contextual factors when investigating psychosocial outcomes of physical activity.

**Support/Funding Source:**

The TwinssCan study received funding from the European Community’s Seventh Framework Program and the Fund of Scientific Research Flanders and Twins. LJ is supported by a PhD Fellowship from Research Foundation Flanders. RvW is supported by a Senior Clinical Fellowship (FWO – 1803616N), and by the Funds Julie Renson, Queen Fabiola and King Baudouin Foundation.

